# PM_2.5_ diminution and haze events over Delhi during the COVID-19 lockdown period: an interplay between the baseline pollution and meteorology

**DOI:** 10.1038/s41598-020-70179-8

**Published:** 2020-08-10

**Authors:** Surendra K. Dhaka, Chetna, Vinay Kumar, Vivek Panwar, A. P. Dimri, Narendra Singh, Prabir K. Patra, Yutaka Matsumi, Masayuki Takigawa, Tomoki Nakayama, Kazuyo Yamaji, Mizuo Kajino, Prakhar Misra, Sachiko Hayashida

**Affiliations:** 1 Radio and Atmospheric Physics Lab, Rajdhani College, University of Delhi, New Delhi, 110015 India; 2 Department of Physics and Astrophysics, University of Delhi, New Delhi, 110007 India; 3 School of Environmental Sciences, Jawaharlal Nehru University, New Delhi, 110067 India; 4 Aryabhatta Research Institute of Observational SciencES (ARIES), Manora Peak, Nainital, 263001 India; 5 Japan Agency for Marine-Earth Science and Technology (JAMSTEC), Yokohama, 2360001 Japan; 6 Institute for Space-Earth Environmental Research, Nagoya University, Nagoya, 4648601 Japan; 7 Faculty of Environmental Science, Nagasaki University, Nagasaki, 8528521 Japan; 8 Graduate School of Maritime Sciences, Kobe University, Kobe, 6580022 Japan; 9 Meteorological Research Institute, Japan Meteorological Agency, Tsukuba, 3050052 Japan; 10 Research Institute for Humanity and Nature, Kyoto, 6038047 Japan; 11 Faculty of Science, Nara Women’s University, Kita-uoya Higashi-machi, Nara, 630-8506 Japan

**Keywords:** Climate sciences, Environmental sciences, Physics

## Abstract

Delhi, a tropical Indian megacity, experiences one of the most severe air pollution in the world, linked with diverse anthropogenic and biomass burning emissions. First phase of COVID-19 lockdown in India, implemented during 25 March to 14 April 2020 resulted in a dramatic near-zeroing of various activities (e.g. traffic, industries, constructions), except the “essential services”. Here, we analysed variations in the fine particulate matter (PM_2.5_) over the Delhi-National Capital Region. Measurements revealed large reductions (by 40–70%) in PM_2.5_ during the first week of lockdown (25–31 March 2020) as compared to the pre-lockdown conditions. However, O_3_ pollution remained high during the lockdown due to non-linear chemistry and dynamics under low aerosol loading. Notably, events of enhanced PM_2.5_ levels (300–400 µg m^−3^) were observed during night and early morning hours in the first week of April after air temperatures fell close to the dew-point (~ 15–17 °C). A haze formation mechanism is suggested through uplifting of fine particles, which is reinforced by condensation of moisture following the sunrise. The study highlights a highly complex interplay between the baseline pollution and meteorology leading to counter intuitive enhancements in pollution, besides an overall improvement in air quality during the COVID-19 lockdown in this part of the world.

## Introduction

The pandemic due to spread of novel Corona virus, commonly known as the COVID-19, has led to partial or complete lockdown in several countries around the world. The spread of deadly virus has caused deaths estimated to more than two hundred thousand people over a period of December 2019–April 2020. However, air pollutants and COVID-19 are linked to have played a major role in huge number of deaths^1,2^. In order to contain its impact in India, the first phase of complete lockdown imposed from 25 March to 14 April 2020, which was further extended till 03 May 2020. As a result, the transport, construction works, industries and other commercial activities, which could have injected pollutants or produce dust, are stopped and remained at its minimal level. Unprecedented reductions in anthropogenic activities yielded to very low values of emissions resulting in significantly improved air quality over the Delhi-National Capital Region (NCR) [up to 50% reduction in fine particle matter of aerodynamic diameter smaller than 2.5 µm (PM_2.5_)]^3–6^. Despite the gloomy and sad day-to-day life and situation in which millions of people are under distress, this period, nevertheless, allowed a unique opportunity for the scientific community to study the interplay between baseline air pollution and natural processes.

Northern Indian winters experience severe widespread air pollution attributed to a variety of emissions being confined near the surface under stagnant metrological conditions^7–9^. Aerosol emissions in India is dominated by transportation, industrial, residential energy usage and biomass burning^10–12^. Present lockdown period, thus, is an opportunity to measure the baseline air pollutants and variability in a natural environment with the least anthropogenic effects. Most of days of the lockdown witnessed clear sky conditions without any signature of visible contamination. These observations are therefore of paramount significance to study and understand the interactions of meteorology and baseline pollution in the tropical megacity. The difference in air quality before and during the lockdown can be used as an estimate of the regional pollution which accumulates on top of the background (levels during lockdown). Such studies are not possible in normal conditions, for example even during the very aggressive “odd–even scheme” the air quality over Delhi was only marginally improved^13,14^, which besides other factors^15,16^, highlighted a remarkable role played by the meteorology and lower atmospheric dynamics.

In light of the above scenarios, continuous measurements of air quality and meteorological parameters have been analyzed to unravel the impact of the lockdown on air quality and to elucidate the interactions between this baseline pollution and meteorology. A peculiar enhancement in PM_2.5_ levels in the early morning and in the night time, even during lockdown, has also been examined. The details of various measurements are described in the data section; followed by deliberations on the observed air quality variations and the underlying processes. Finally, the salient features and perspectives of this work are presented in the discussion section.

## Methods and data

Observations of PM_2.5_ were carried out using Compact and Useful PM_2.5_ Instrument (CUPI); this is a compact size smart Panasonic sensor along with data logger (hereinafter called CUPI)^17^. This sensor estimates total volume of the PM_2.5_ by measuring counts and intensities of scattering signals from single particles. The total volume of the PM_2.5_ was converted to mass concentration using a conversion factor of 1.4, as was determined in our previous study^18^. Unit-to-unit variation of the sensitivity of the sensor is typically less than 10% and accuracy of the sensor is typically less than 23% at relatively high temporal resolution of < 2 min^17^.

**Figure 1:**
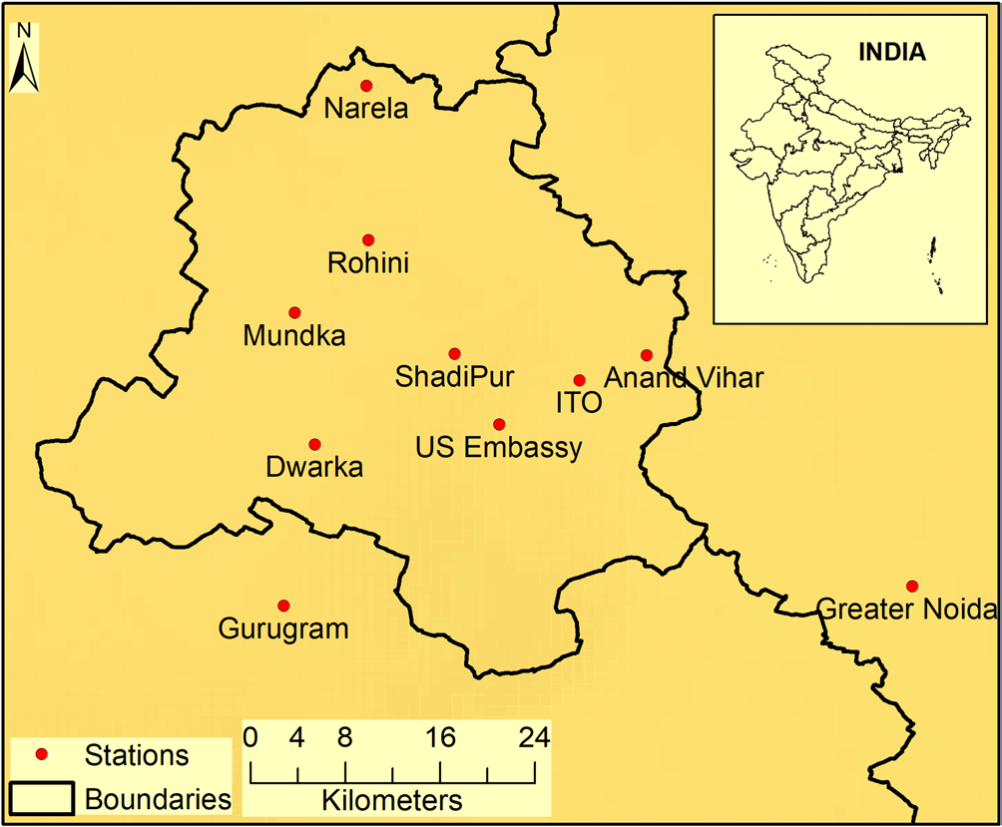
Locations of the stations are shown on the Map of Delhi, Two stations [Gurugram and Greater Noida represent part of National Capital Region (NCR)]. Stations represent both outskirt and center of the city. Map is generated using software ArcGIS 10.3.

Data collected at eight monitoring stations in the Delhi-NCR during 1 March to 14 April, 2020 have also been analyzed. Different locations of eight monitoring stations are shown over Delhi map in Fig. 1, which fairly represent all regions of the city. In addition, solar radiation, wind, rainfall, relative humidity and temperature observations are examined to present meteorological conditions. The spatial coverage usually represents significant variability depending upon low to high emissions, in normal days, from one specific location to the other. Here diurnal and averaged variation of PM_2.5_ from three monitoring sources viz., (1) using CUPI, that provides data with high temporal resolution; (2) Delhi Pollution Control Committee (DPCC), and (3) Central Pollution Control Board (CPCB) during and prior to this lockdown is presented. CPCB provides data quality assurance by following rigorous protocols for the sampling, analysis and calibration. Continuous ambient air quality monitoring (CAAQM) systems are used. Multipoint calibration using automatic dilution system for the calibration or/and auto calibration are in practice. Meteorological instruments precisely measure with high accuracy and resolutions of all the atmospheric parameters such as wind speed, wind direction, ambient temperature, relative humidity, solar radiation, and atmospheric pressure using state of the art technology, which involves automated transfer of data every 15 min.

Observations at high temporal resolution using CUPI are used from 1 to 14 April, 2020. CUPI is located in sector 4, Dwarka at about 4 km away from DPCC Dwarka monitoring site. DPCC and CPCB monitoring stations provide data at the time resolutions of 15 min. Observations are analyzed to delineate the difference of air quality changes between prior and during lockdown periods.

## Results

The effects of lockdown on the atmospheric measurements are analyzed over the Delhi-NCR as compared to the reference conditions i.e., prior the lockdown, as presented in the following subsections.

### General features prior and during lockdown

**Figure 2:**
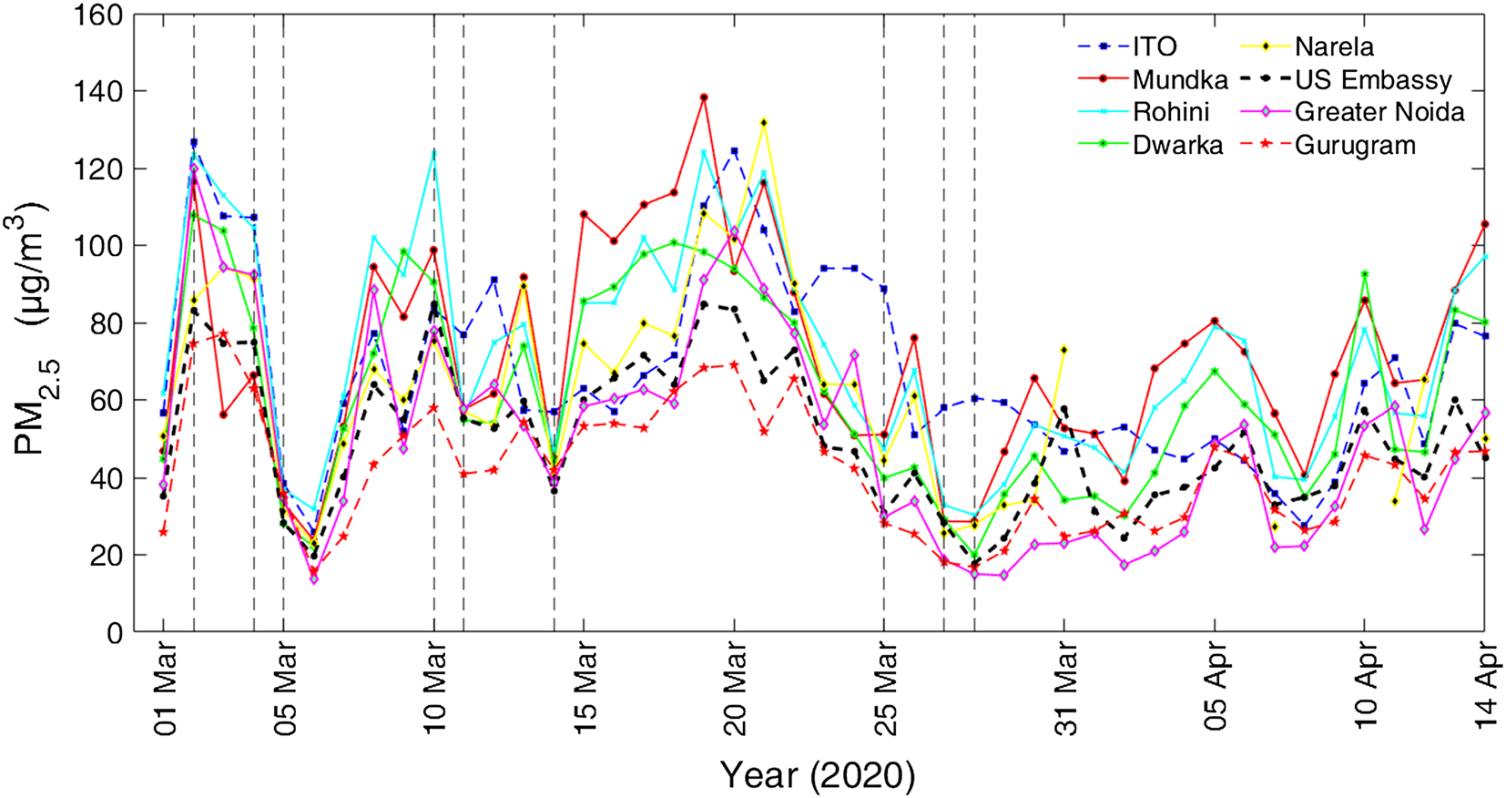
Daily averaged PM_2.5_ concentration from 1 March to 14 April 2020 (covering first phase of lockdown period from 25 March to 14 April) over eight stations (ITO, Mundka, Rohini, Dwarka, Narela, US Embassy, Greater Noida and Gurugram). In addition, rainfall occurrence days are shown with vertical dashed lines, which correspond to dip in the PM_2.5_. Rainfall was relatively larger on 4–5 March 2020 (~ 15–20 mm) and 14 March 2020 (~ 40 mm) in comparison to other days.

Figure 2 illustrates daily averaged PM_2.5_ values among the chosen sites during 01 March–14 April, 2020. Daily mean values of PM_2.5_ concentrations show similar variations and range from 15–130 µg m^−3^ with peak values of 70–120 µg m^−3^. Sharp diminutions in PM_2.5_ during 3–5 March, 14 March and then later on 27–28 March 2020 correspond to rain occurrences (marked by vertical dotted blue lines). Rain washout effect led to decrease of less than 30 µg m^−3^ (~ 50 µg m^−3^) around 3–5 March (and 14 March). During lockdown (25 March–14 April, 2020) there was a substantial decrease (50 ± 15%) in PM_2.5_ in all the three weeks, however a quasi-periodic behavior of 5–6 days with a tendency of linear increase in PM_2.5_ is shown in second and third week.

Prior and during first week of lockdown, the decreases in PM_2.5_ concentrations range from ~ 70 to 40% as seen from the observations at 9 stations (Supplementary Fig. S1). During lockdown, PM_2.5_ remained within a range of ~ 35–40 µg m^−3^ in the ambient atmosphere, which is amazingly low in Delhi-NCR as far as the air quality over the past two decades is concerned^15^. Supplementary Fig. S2 depicts similar characteristic of averaged (eight stations data) diurnal variations of PM_2.5_ during prior and lockdown period. Permanent features of morning and evening peaks associated with boundary layer dynamics remained distinctly similar though scaling during lockdown is reduced. During lockdown, the peak PM_2.5_ concentrations (~ 20–30 µg m^−3^) are slightly lower in the morning and late night. This implies that local meteorology and boundary layer dynamics control the diurnal variation of PM_2.5_. It is worth noticing that during the early morning and late night, wind speed is very low (< 1 m s^−1^) and temperature remains around 20 °C or less, which are favorable conditions for non-dispersion of pollutants. These low temperature and stagnation of wind movement in the absence of solar radiation also contribute to the formation of haze (unactivated particles consisting both of liquid water and other compounds by hygroscopic growth) and possibly mist (activated larger particles mainly consisting of liquid water). Despite the fact of having no source of anthropogenic emission, we could not find any change in the characteristic feature of diurnal variability of PM_2.5_. Only some of the days showing almost constant values at all hours, which were without haze in the morning and observed moderate wind speed.

Moreover, PM_10_ concentrations were also observed to reduce substantially, by a factor of 2–4, and attaining less than 80 µg m^−3^ (Supplementary Fig. S3a), which is similar to behaviour of PM_2.5_ variations. During lockdown the air quality has improved considerably and approached to the prescribed safe limit of World Health Organization (WHO)^19^ standards of 25 µg m^−3^ for PM_2.5_, and Indian National Ambient Air Quality Standard (NAAQS) for PM_2.5_ of 40 µg m^−3^. It is worth mentioning here that several other gaseous species resulting from emissions of road transport, biomass burning, and factories, viz., SO_2_, CO, ammonia and NO_2_ were also reduced by an average factor of about 2–3 (shown in Supplementary Fig. S3b–f). In contrast, the photochemically produced O_3_ showed an increased level (Supplementary Fig. S3d). The O_3_ enhancement could be attributed to an increase in the solar insolation by allowing more radiations to reach on earth’s surface leading to faster formation rate of O_3_ in clean atmospheric conditions, decrease in heterogeneous loss of HO_2_ due to reduction of aerosol particles, and decrease in titration reaction of O_3_ by NO due to less vehicular emission. For brevity, dynamics and/or chemistry of the gases variations are not discussed in detailed as our analysis is limited to PM_2.5_ variations during prior and lockdown.

### High resolution observations of PM_2.5_ and meteorological conditions

**Figure 3:**
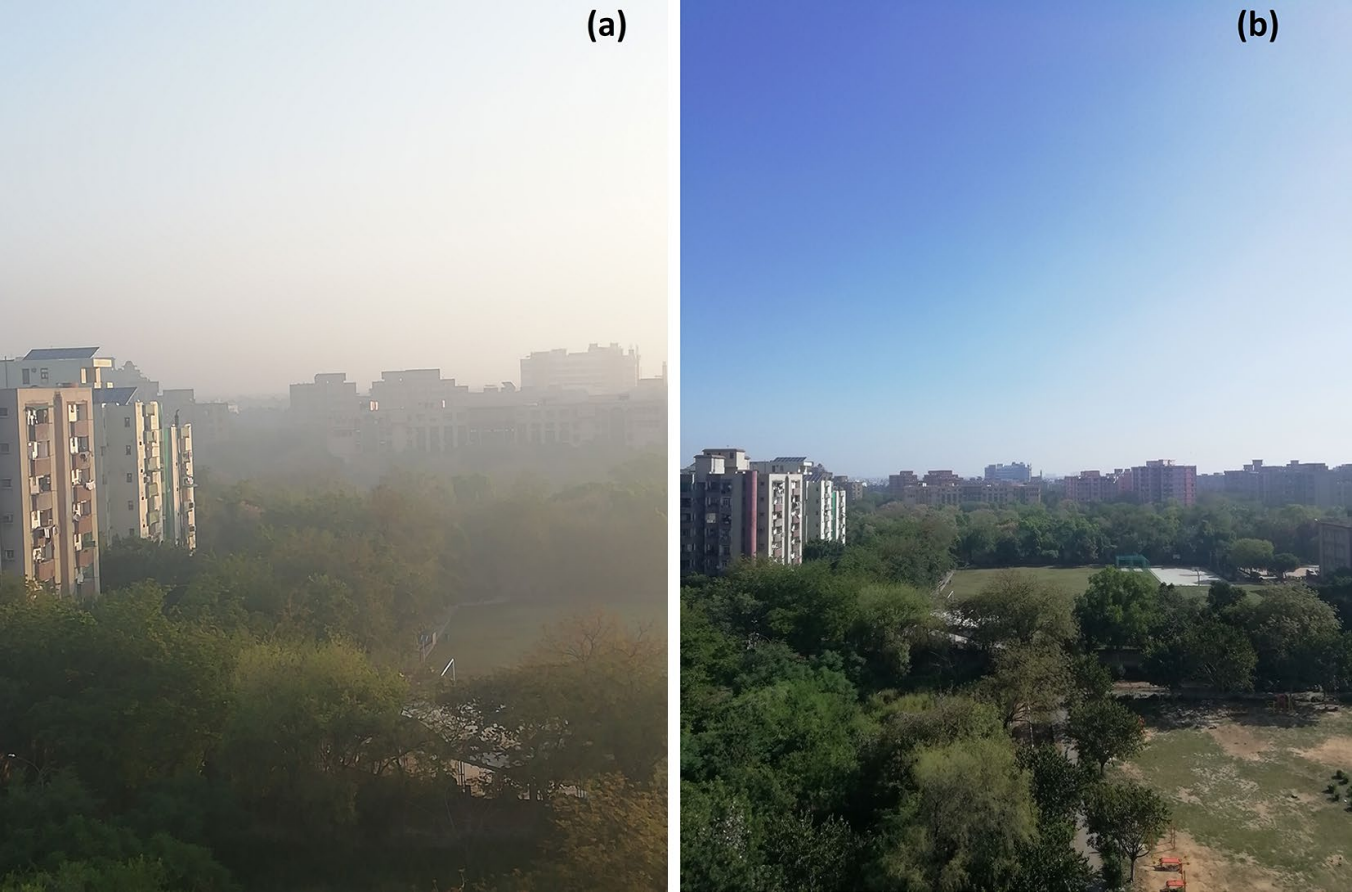
(**a**) A typical example of early morning haze at 0700 IST (left) and (**b**), clear sky at 1000 IST on 03 April 2020 (right). Sun rise was at 0610 IST. Haze disseminated within 3 h. Blue and clear sky conditions remained until 7–8 April 2020 and then bit dusty environment prevailed for 3rd week of April 2000.

In the naturally controlled environment, intriguing relation among different atmospheric parameters with mechanism of rise in PM_2.5_ in morning hours in the presence of visible haze (Fig. 3a, left panel, which gets dissipated just after sunrise, Fig. 3b, right panel) is investigated. In general, morning and late evening hours witness higher concentration of PM_2.5_. Usually, primary peak occurred in morning (0700–1000 IST) and secondary peak after 2100 IST (which was augmented mainly due to vehicular emissions; Supplementary Fig. S2).

**Figure 4:**
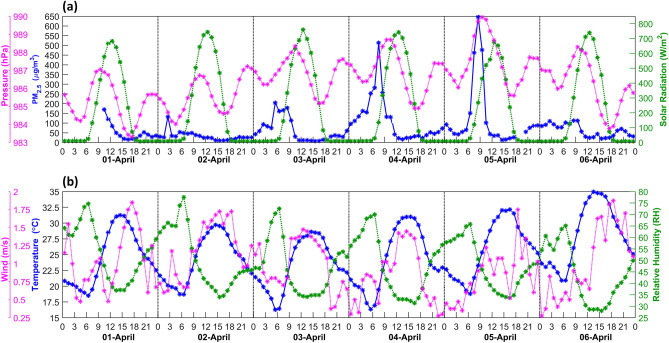
Upper panel (**a**) Hourly averaged PM_2.5_ concentration based on Compact Useful Particle Instrument (CUPI) monitoring at Dwarka. Solar radiation (W m^−2^), barometric pressure (hPa) are also shown. Lower panel (**b**) illustrates temperature (°C), relative humidity (%) and wind speed (m s^−1^). Rest all other fields are monitored at Dwarka DPCC station (~ 3 km away from CUPI monitoring point) and shown during second week of locked down period (from 1 to 6 April 2020).

As mentioned earlier, concentration of particulate matter reduced considerably in the first week of lockdown and brought down the peak value to about ~ 40 µg m^−3^. Diurnal variation of PM_2.5_ (hourly mean) with pressure and solar radiation is shown in Fig. 4a; and for temperature, relative humidity and wind speed in Fig. 4b. Data are shown for 1 April to 6 April 2020 only. It is pertinent to mention here that at a fine temporal scale, there is a large variability in PM_2.5_ (peaking up to 100–200 µg m^−3^ and even touching ~ 400–500 µg m^−3^ on some days) in morning around 0600–1000 IST and then reduced to ~ 40 µg m^−3^ throughout day.

The feature of high PM_2.5_ in the morning hours along with the formation of visible haze conditions is a major concern of this study. Occurrences of high PM_2.5_ primarily in the absence of large contaminations from vehicular and anthropogenic emissions but in the presence of hygroscopic fine particles in a naturally controlled environment are intriguing. The suggested mechanism for the same could be due to the presence of fine mode (< 1 µm; PM_1_) particles near the surface which grow with the availability of ample moisture content in the atmosphere close to surface (within the canopy height) on a given day. This, after the sunrise starts mixing due to boundary layer processes and commissions the haze. It is important to mention here that there is a marked variation in the formation of haze depending upon the location of measuring site whether it is in mid-city or sub-urban region of Delhi-NCR. During day, however, there is fair similarity of observed PM_2.5_.

Gani et al.^20^ have observed marked seasonal and diurnal variability in the concentration and composition of PM_1_. They discussed that such features emerged owing to the interactions of sources and atmospheric processes. During March–April, averaged PM_1_ remains in Delhi in the range of ∼ 100 µg m^−3^, being highest during winter ∼ 210 µg m^−3^. In addition, the contributions of the secondary species were observed even larger than the primary emissions. Higher concentrations of sub-micron particles seem to be playing a significant role in the formation of haze in the morning hours. With the rising temperature and mixing processes in the boundary layer during initial 2–3 h of solar radiation, these fine particles, which are likely to act as condensation nuclei, undergo hygroscopic growth and attain the size of PM_2.5_ matter. In the most recent and significant study by Wang and Chen^21^ reported high hygroscopicity of aerosol particles in Delhi. This nature of particles could be crucial for the formation of high concentration of PM_2.5_. It is also accepted that aerosols mixed/coated with black carbons inhibits hygroscopic particle growth^22,23^. These particles are considered to play a key role in the formation of mist/haze droplets. It may also lead to the formation of larger particles due to coalescing. Since the CUPI is sensitive to both liquid water and other compositions in the particles with a diameter less than 2.5 μm under high relative humidity conditions (typically, > 70%) as shown by Nakayama et al.^17^, and shoots up the observed concentrations in the morning hours as the haze is lifted to sensor height of ~ 30 m. In addition, Fig. 4 show that the low temperatures and slightly higher moisture content on the previous day might have also led to the condensation of moisture at surface of the earth, grass and tree leaves, which in turn on the next morning helps forming the haze particles in the process of mixing. Whereas we do not have information on the particle size, a fraction of haze particles would be further activated to form mist or fog droplets during uplifting.

Further, the variations in pressure, solar radiation, temperature and wind speed are examined to understand the physical process of the peculiar haze conditions. In general, there is out of phase relation between temperature and relative humidity at Dwarka DPCC site. However, temperature and wind are having similar variability, sometimes with a lag or lead. Decreased temperature and low wind with increased relative humidity under these changed atmospheric conditions led to condensation and the haze formation in the night time hours. However, the morning episode of haze is caused by the vertical lifting of moisture from surface, tree leaves, and leading to coalescing processes as a result of mixing. Semi-diurnal variation with primary peak in morning around 0900–1000 IST also shows close association of PM_2.5_ with pressure and solar radiation. And a secondary peak during night (~ 2100–2400 IST) is observed. The pressure difference between primary and secondary peaks remains in the order of ~ 1–2 hPa. It is interesting to note that this diurnal difference of ~ 1–2 hPa prevails even during slowly rising pressure over the week which increased by ~ 6 hPa. Slowly rising pressure corresponds to high values in PM_2.5_ in morning during 3–5 April 2020. In the low wind conditions, local pressure variation, under the given ideal circumstances during morning and late night, apparently controls diurnal variability of PM_2.5_ (Fig. 4b). Solar semi-diurnal tidal components need to be re-examined to understand variability of PM_2.5_ as it is linked with wind speed and variability over land and ocean regions and eventually atmospheric stability and formation of fog^24^.

Increased (decreased) wind conditions provide dynamical stability for disbursing (accumulating) the PM_2.5_. On the other hand, mixing layer height (boundary layer) rises during spring after 0900 IST and sinks during morning and night^15^. However, it does not seem to be so sensitive to reflect changes in slowly growing pressure. Role of diurnal temperature variation, Fig. 4b, also corresponds with distribution of PM_2.5_ and relative humidity as well.

Further, as Fig. 4a illustrates hourly averaged PM_2.5_ peaks appearing in morning for about 3–4 h, visible haze (Fig. 3a) stayed until 1000–1100 IST. As soon as the radiation peaks up by 1000 IST onwards, there is a clear indication of evaporation of tiny droplets of haze (Fig. 3b). For instance, on 02 April 2020 it was a clear sky with very low PM_2.5_ around 15–20 µg m^−3^ in the afternoon. With the intensified solar radiation around 0930–1030 IST (from 200 to 500 W m^−2^) and wind increasing at ~ 2 m s^−1^, there is a sudden decrease in the PM_2.5_ within an hour up to 20 µg m^−3^. Present case during 1–6 April 2020 provided us an opportunity to estimate the contribution coming from hygroscopic growth of fine mode particles.

Accumulation of PM_2.5_, due to natural conditions in the presence of visible haze coinciding with the peak semi-diurnal pressure, amounts to be in the range of 50–200 µg m^−3^. Note that late night secondary peak of semi-diurnal pressure corresponds with PM_2.5_ and remains around 50–75 µg m^−3^ on 3–5 April 2020 in the absence of night time condensation resulting into haze. Tendency of slight increase in PM_2.5_ is also noted with gradual increase in the temperature and decrease in relative humidity during 2nd–3rd week. By the end of 3rd week of lockdown (from 11 to 14 April 2020), increase in PM_2.5_ is seen with temperature already notching up to 41 °C, relative humidity declining to 50% with reducing wind speed. Vertical atmosphere is no more that cleaner and a dusty environment prevails. Therefore, first week of April 2020 is an ideal for background conditions to investigate the relationship of several atmospheric parameters.

**Figure 5:**
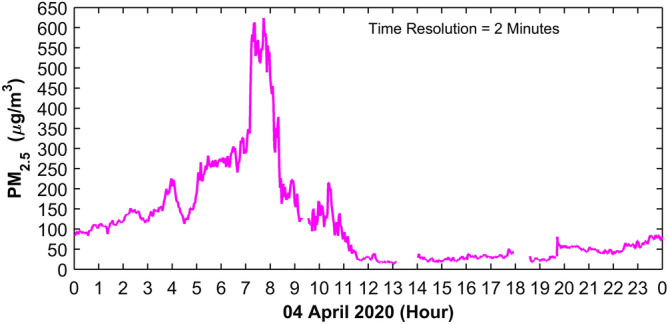
High resolution monitoring (averaged over 2 min interval) of PM_2.5_ shown since morning to evening. On 4 April 2020 from 0500 to 0700 IST haze prevailed. After sunrise, around 0710 IST, within 20 min (0730 IST onwards) further a steep and step increase of about 100–300 µg m^−3^ in PM_2.5_ took place, which lasted for about an hour. Steep and step wise increase was a common feature for several days. As soon as solar radiation increased above 300 W m^−2^, and wind speed increased around 1000 IST, sudden decline in PM_2.5_ observed up to 20 µg m^−3^.

Further using fine temporal scale data sudden changes in PM_2.5_ especially in the morning, while visible haze was present and sun rise was taking place (solar radiation just started), is examined. As an example, the case of 4 April 2020 is presented in Fig. 5. From 0530–0730 IST, haze prevailed and PM_2.5_ was observed to be in the range 200–300 µg m^−3^. After half an hour of sun rise around 0730 IST, sudden increase in PM_2.5_ was observed, and after every minute the increase was in the range 10–20 µg m^−3^ per minute. Interestingly, within 10 min PM_2.5_ increased by 100 µg m^−3^ for about an hour (i.e., until 0830 IST). In the meantime solar radiation and wind speed were peaking up, temperature increased nearly 25 °C, and then there was a sudden decline in PM_2.5_ (~ 20 µg m^−3^) after 1000 IST. Thus in this process, PM_2.5_ distinctly increased stepwise by 0830 IST and then decreased in the similar manner until 1000 IST. During early morning hours temperatures were within the range of ~ 15–17 °C, quite close to the dew point (1–6 April 2020), led the mechanism for haze formation which becomes conducive for consistent increase in PM_2.5_ concentration for next 3–4 h.

After sun rise, a new mechanism involving mild convection and evaporation of existing moisture operates and therefore concentration becomes higher by a factor of about 1.5 for a duration of about an hour. On everyday basis, physical examination found enough dew on the leaves and grass around the observation site. Rajput et al.^25^ suggested that organic aerosols (especially aged biomass burning factor) are enhanced by fog-processing in winter at Kanpur. Also, Eck et al.^26^ suggested that particle size (of fine mode particles) increased after the fog events at Kanpur. Current observations confirm the increase in mass concentration of PM_2.5_ even in the pristine environment.

In contrast with several directly-emitted chemical species, the O_3_ pollution at all the stations in NCR showed variability and magnitudes during the lockdown similar to those during pre-lockdown conditions, however with the rising tendency (Supplementary Fig. S3). This is suggested to be due to non-linear chemistry enhancing O_3_ when NOx is reduced (Supplementary Fig. S3–f) in VOC-limited chemical regime of Delhi^27–29^. A simultaneous reduction in PM_2.5_ would have also contributed to this increase in O_3_ attributed to the effects of aerosols on photolysis^7,29,30^.

## Discussions

Continuous observations from eight locations in Delhi-NCR using data from CPCB, DPCC and CUPI have shown a clear indication of decrease in PM_2.5_ in 3 weeks of lockdown (25 March–14 April, 2020). Reduction in emission is found in the range of 40–70% depending upon the location. Baseline measurement of PM_2.5_ in the naturally controlled environment led to the formation of haze in morning hours and the considerable increase in PM_2.5_ is investigated especially during first and second weeks of lockdown. During day, a fairly low PM_2.5_ was observed, which lied in the range of 20–40 µg m^−3^.

A profound steep rise in PM_2.5_, after sunrise, is an exciting finding which was observed almost all days in the first week of April 2020 suggesting evaporation of moisture from ground and trees contributing to the density of particles in the diameter range ~ 2.5 micron. The episodic amount of about 150–200 µg m^−3^ in the morning hours was not produced by burning, transport, industry, and construction. In an ideal condition of low wind, temperature, and planetary boundary layer dynamics, along with peak of the cycle of semi-diurnal variation in local pressure, are very favorable in enhancing the fluctuation of PM_2.5_ in the morning during lockdown. High resolution observations using CUPI during lockdown has shown this intriguing feature of unusually high PM_2.5_ in clear sky conditions. It is important to mention here that number density of PM_2.5_ can be a mix of pure particles and water droplets of similar size up to some extent, for which CUPI is not segregating.

It is investigated from these observations that even in the clear sky conditions, haze formation can develop substantial amount of PM_2.5_. These tiny droplets of haze (or mist) evaporate quickly with the rise in solar radiation in a time period of 2–3 h. As soon as radiation reached in the range of 300–500 W m^−2^ a rapid decline in PM_2.5_ concentration was seen, also wind speed peaked up to ~ 2–3 m s^−1^ in the afternoon that made a clear sky within 2 h.

Covid-19 lockdown has provided us a nearly natural laboratory to investigate the close association of haze and development of PM_2.5_ that amounted to be around 100–150 µg m^−3^ while rest of the day background PM_2.5_ remains as low as low as 15–20 µg m^−3^, which is unprecedented situation in Delhi-NCR, Northern India. This study opens up a microphysical regime to be investigated further in the context of subtle atmospheric processes operating in natural environment in conjunction with intensive measurements available on environmental issues.

## Supplementary information

Supplementary Figure S1.

Supplementary Figure S2.

Supplementary Figure S3.

Supplementary Legends.
