# Supplementary figures and images for: PM2.5 diminution and haze events over Delhi during the COVID-19 lockdown period: an interplay between the baseline pollution and meteorology
Source: Sci Rep. 2020 Aug 10;10:13442. doi: 10.1038/s41598-020-70179-8 (PMC7417527; doi:10.1038/s41598-020-70179-8)

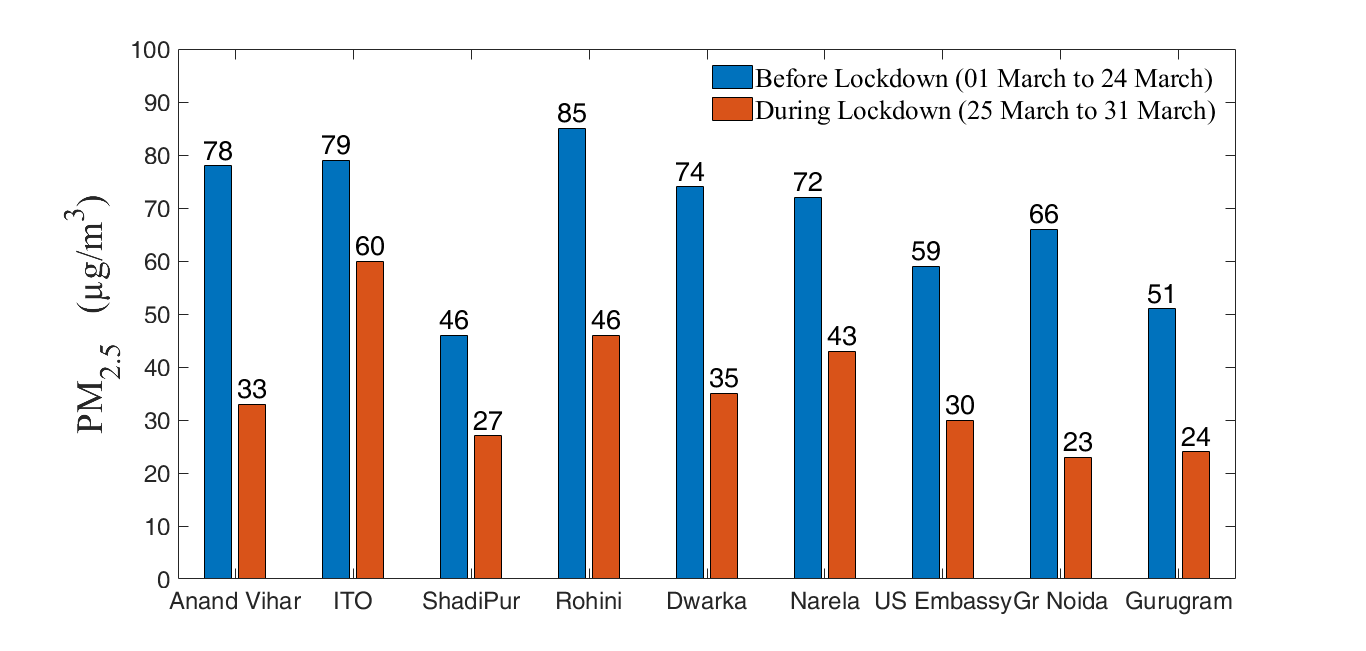

Supplement: Supplementary file 1 — Supplementary Figure S1. [file 41598_2020_70179_MOESM1_ESM.tif]

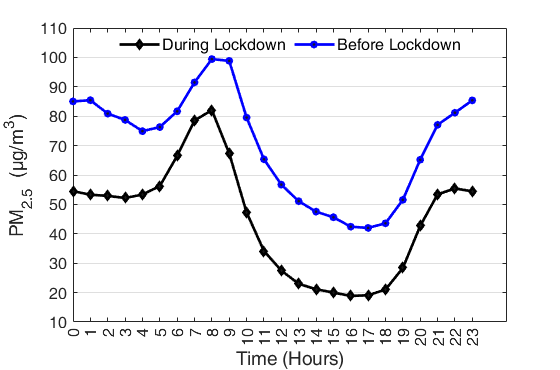

Supplement: Supplementary file 2 — Supplementary Figure S2. [file 41598_2020_70179_MOESM2_ESM.tif]

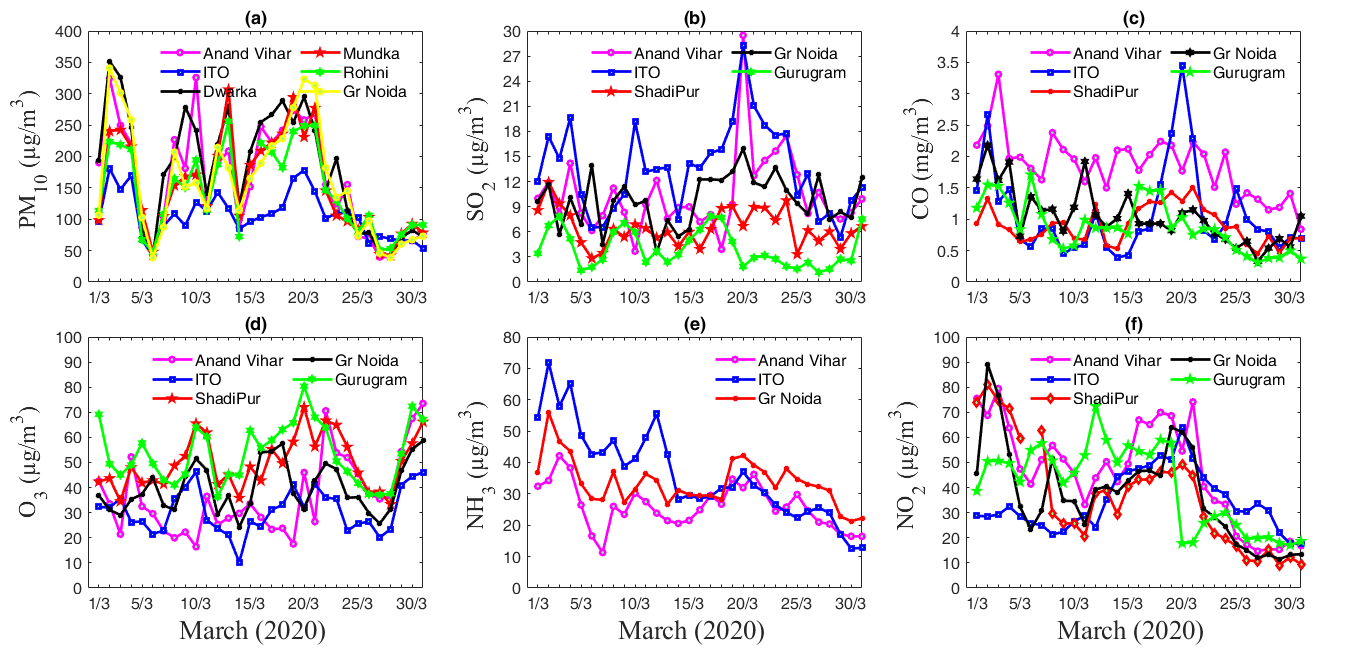

Supplement: Supplementary file 3 — Supplementary Figure S3. [file 41598_2020_70179_MOESM3_ESM.tif]
