# Supplementary material for: PM2.5 diminution and haze events over Delhi during the COVID-19 lockdown period: an interplay between the baseline pollution and meteorology
Source: Sci Rep. 2020 Aug 10;10:13442. doi: 10.1038/s41598-020-70179-8 (PMC7417527; doi:10.1038/s41598-020-70179-8)
Supplement: Supplementary file 4 — Supplementary Legends. [file 41598_2020_70179_MOESM4_ESM.docx]

Fig. S1. Description of PM_2.5_ averaged concentrations on 8 sites in Delhi-NCR before lockdown (1 March to 24 March 2020: blue color) and during lockdown (25 March to 31 March 2020: red colors) using DPCC and CPCB monitoring observations during 25^th^ – 31^st^ March 2020. A clear decline in the concentrations is seen during last week due to locked down period in the wake of COVID-19. During April 2020, PM_2.5_ remains low, though having a tendency of linear increase with quasi-periodic fluctuations as seen in Fig. 1a.

Fig. S2. Diurnal variation averaged at all 8 stations before lockdown (1 March to 24 March 2020: blue color) and during lockdown (25 March to 14 April 2020: black color) period over Delhi-NCR. Both the curves follow similar tendency of rising in the morning and evening, however, from 1000 IST to 2000 IST it remains less than 40 µg m^-3^. Twin peaks behavior seems a natural tendency of PM_2.5_ in Delhi-NCR.

Fig. S3. Description of (a) PM_10_, (b) SO_2_, (c) CO, (d) O_3_, (e) Ammonia and (f) NO_2_ concentrations/emissions on prominent sites in Delhi-NCR (marked within the panel in different colors) using DPCC and CPCB monitoring observations during March 2020. A clear decline in the PM_10_ and gaseous emissions of different species are observed, except that of O_3_ which showed increase and SO_2_remains almost constant during first week of lockdown
